# Supplementary material for: Combined Strategies for Improving Aflatoxin B1 Degradation Ability and Yield of a Bacillus licheniformis CotA-Laccase
Source: Int J Mol Sci. 2024 Jun 12;25(12):6455. doi: 10.3390/ijms25126455 (PMC11203865; doi:10.3390/ijms25126455)
Supplement: Supplementary file 1 [file ijms-25-06455-s001.zip › ijms-3055269-Supplementary.pdf]

Supplementary data

Table S1. Catalytic activity for the WT and mutants using ABTS as substrate.

**CotA-laccase**	**Catalytic activity (U/L)**
WT	55.12
E186A	171.38
E186R	210.74
E186K	22.41
F205Y	7.33
R208G	196.38
K315N	111.96
G322S	15.90
A376I	101.72
L385W	329.62
I417G	30.80
P454S	9.74
D500G	62.56

Table S2. Primers used in this study.

Gene	Primer	Sequence (5′ to 3′) [1]
E186A	*E186A*-F	TTGAACTTGCCGAAGGGAGCATATGAAATC
	E186A-R	GCTCCCTTCGGCAAGTTCAATGAACGCTCT
E186R	*E186R*-F	TTGAACTTGCCGAAGGGACGATATGAAATC
	E186R-R	CGTCCCTTCGGCAAGTTCAATGAACGCTCT
E186K	*E186K*-F	ATTGAACTTGCCGAAGGGAAAATATGAAAT
	E186K-R	TTCCCTTCGGCAAGTTCAATGAACGCTCTT
F205Y	*F205Y*-F	CATGAAGATGGTTCATTGTATTATCCGCGG
	F205Y-R	TACAATGAACCATCTTCATGAAATGATTTA
R208G	*R208G*-F	TGGTTCATTGTTTTATCCGGGGCAGCCTGA
	R208G-R	CCGGATAAAACAATGAACCATCTTCATGAA
K315N	*K315N*-F	GAAAAACCGTGACACTGAACAACCGGATCG
	K315N-R	GTTCAGTGTCACGGTTTTTCCTTCTGCTCG
G322S	*G322S*-F	AACCGGATCGGCTGCGGCTCACAAGACGCA
	G322S-R	GAGCCGCAGCCGATCCGGTTTTTCAGTGTC
A376I	*A376I*-F	AATCTGTCATTGGGCGCGATCGTTGACCAA
	A376I-R	ATCGCGCCCAATGACAGATTTCTGAGGGCA
L385W	*L385W*-F	CAATATGGAAGACCTGTTTGGCTTTTAAAC
	L385W-R	CAAACAGGTCTTCCATATTGGTCAACGGCC
I417G	*I417G*-F	ATCAATGCCGGAAGGGCGGGCCATCCGATC
	I417G-R	CCCGCCCTTCCGGCATTGATGATCGACCAG
P454S	*P454S*-F	TTTTACCGGTCCGGCAGTTTCTCCGGCACC
	P454S-R	AAACTGCCGGACCGGTAAAACAAGTTCTC
D500G	*D500G*-F	CTTGAGCACGAAGATTACGGTATGATGCGC
	D500G-R	CCGTAATCTTCGTGCTCAAGGATGTGGCAG

[1] The underlined sequences indicate mutant sites and cloning sites.

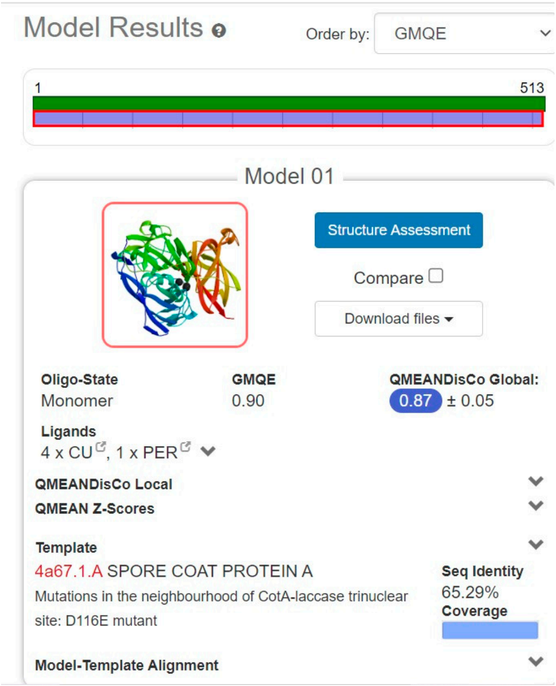

Figure S1. Homologous modeling of CotA-laccase from *Bacillus licheniformis* ANSB821.

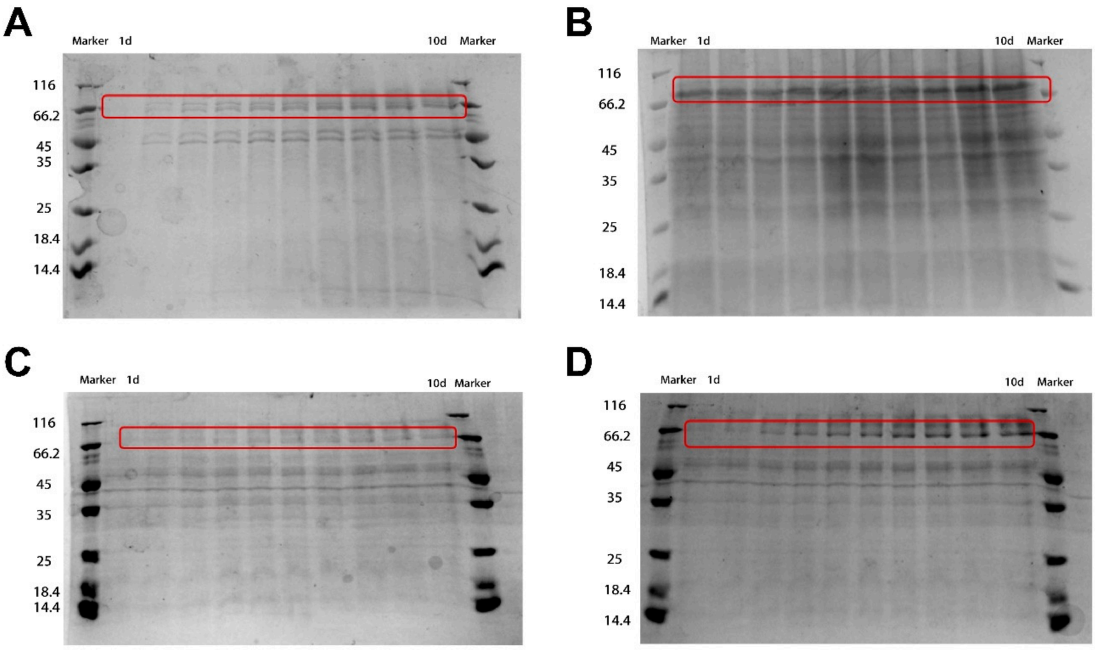

Figure S2. SDS-PAGE of the fermentation supernatant of GS115-pPICZαA-WT (A), GS115-pPICZαA-N-WT (B), GS115-pPICZαA-N-E186A (C), and GS115-pPICZαA-N-E186R (D) cultured for 1-10 d.
